# Exploring piling behavior in aviary-housed laying hens in response to age, management practices, and social influences

**DOI:** 10.1016/j.psj.2026.107961

**Published:** 2026-10-17

**Authors:** Valentina Bongiorno, Ahmed B.A. Ali, Janice M. Siegford

**Affiliations:** a Department of Animal Science, Michigan State University, 474 S. Shaw Lane, East Lansing, Michigan, 48824-1225, United States of America; b Department of Animal and Veterinary Sciences, Clemson University, Clemson, SC, 29634, United States of America; c Animal Behavior and Management, Department of Veterinary Hygiene, Veterinary Medicine, Cairo University, Cairo, 12613, Egypt

**Keywords:** Laying hen, Cage-free system, Poultry welfare, Animal behavior

## Abstract

Piling behavior (PB) represents a poorly understood although relevant economic and welfare issue in laying hens housed in cage-free systems. The current study described possible social facilitation effects on PB using video recordings collected at 17, 27, and 44 weeks of age (WOA) in partial or continuous litter-access aviaries. A flock of 1152 Hy-line brown laying hens were allotted to 2 rooms each equipped with 4 units (144 birds/unit) separated by wire mesh partitions. Hens in one room had continuous litter access, while in the other room birds were kept off litter from lights on until 1200 between 17 and 32 WOA. Evaluation of PB was performed using video footage collected over 48 h at 17, 27, and 44 WOA. Descriptive statistics regarding pile characteristics (number of piles, dimension, duration, preceding events observed, birds involved/total on litter, and activity of adjacent units) were performed. A Monte Carlo analysis was applied to explore possible social facilitation. A total of 95 piling events lasting from 1.25 to 65.67 min were recorded and involved 10 to 69 birds; most of the piles involved over 50% and up to 100% of birds on litter. No piling events were observed at 17 WOA. More piling events were observed in control (CON) hens at 27 WOA than partially enclosed (PARR) birds (30*vs*19), suggesting that time available on litter may influence PB frequency. Furthermore, more preceding events were identified at 44 WOA than at 27 WOA, despite an overall similar number of piling events. The start of a pile in one unit generally did not relate to changes in behavior in neighboring units; furthermore, the results of the Monte Carlo simulation pointed towards the absence of social facilitation, as the clusters of piling events were not greater than what would be observable by chance. The present study provides preliminary insights into PB occurrence in relation to a floor egg management strategy across different ages.

## Introduction

Behavior expression is driven by biological motivation and adjustments to the specific environmental context and can be either a cause or consequence of welfare and production issues (Dellmeier, 1989). However, the common thread linking behavior, welfare, and production may not be always clearly identifiable, and determining the direction in which these factors influence each other can be even more difficult. Broom and Fraser (2007), described abnormal behaviors as the ones that *differ in pattern, frequency or context from that which is shown by most members of the species in conditions that allow a full range of behavior.* In rare circumstances they may represent functional behaviors (e.g. some courtship and parental behavior) with positive connotation; however, in most cases are directly related to unfavorable environmental conditions (e.g. pathologies, life threatening situations) or can be performed as consequential copying mechanism, representing an animal welfare concern (Broom, 2019). Abnormal behaviors are well documented in several species, ranging from laboratory animals (Polanco et al., 2020) to farmed animals (Brunberg et al., 2016). Such behaviors originate from biological needs that animals are unable to fulfill, and examples in commercial poultry production (e.g. feather pecking, toe pecking, piling) have been reported before (Gebhardt-Henrich et al., 2023; Gray et al., 2020; Zepp et al., 2018; Hartcher et al., 2016).

Piling behavior (**PB**) represents a detrimental phenomenon occurring in poultry belonging to the category of abnormal behaviors and it has been documented consistently for more than a decade in Europe and the US in laying hens (Bestman and Wagenaar, 2014; Campbell et al., 2016; Grafl et al., 2017; Winter et al., 2021, 2022b), turkeys (EFSA, 2026), and broiler chickens (Leishman and Riber, 2025). PB consists of birds gathering in dense clusters on litter (Bright and Johnson, 2011), and in some cases it can be triggered by known causes such as laying site preference (nest boxes piling) or fearful stimuli (panic piling). However, in many cases there are no clearly identifiable preceding events (recurring or creeping piling) (Gray et al., 2020).

Overall, PB descried in various studies varies in terms of pile size, duration, and frequency, with no straightforward connection to strain and time in the birds’ life cycle (Armstrong et al., 2023; Campbell et al., 2016; Herbert et al., 2021; Winter et al., 2021; Winter et al., 2022a,b). Moreover, environmental characteristics, like the presence of corners, and routine management practices (e.g., presence of stock person walking across the barn that can capture bird interest) may affect piling occurrence (Gray et al., 2020). Among these practices, floor-egg management such as restricting birds access to litter during the morning, when peak egg laying occurs (as described by Villanueva et al., 2017; Oliveira et al., 2019) was found to negatively affect wing flapping and dust bathing performance (Bongiorno et al., 2026).

Research showed that the simultaneous performance of some behaviors is common in domestic fowl, from feeding (Collins et al., 2011), to nesting (Giersberg et al., 2019), dustbathing (Duncan, 1998), and resting (Riber et al., 2007). PB may be socially facilitated (Gray et al., 2020; Winter et al., 2021) although the potential impact of other birds piling near ongoing piles has not been evaluated. Social facilitation identifies a situation where the behavior performed by one individual releases the performance of the same behavior in another individual and can be driven by increased attention towards a stimulus or location (Nicol, 1995).

PB represents a frequent phenomenon in cage-free laying hens; it raises relevant economic and welfare issues due to smothering occurrences, reported by farmers in UK to account for 2.5% - 38.4% annual flock mortality (Winter et al., 2022), and accounts for industry losses up to £6.5 million/year (Herbert et al., 2021). Moreover, a reduction of 7.5 eggs/1000 birds per day was reported in previous research, despite a positive association found between egg quality and pile duration (Armstrong et al., 2023). Finally, even when no smothering occurs, possible heat and physiological distress underline the disruptive effect of PB on animal welfare (Gray et al., 2020). Describing if and how piles influence each other and how they can change across different management practices can help us understand the causes of PB and how to correct or prevent it.

The current experimental design originates from a broader research project that included the evaluation of different strategies, specifically litter access restriction, to limit floor eggs. Litter access restriction represents an interesting factor to investigate within poultry behavior research, as the expression of natural positive behaviors of the birds can be compromised (Bongiorno et al., 2026) and an increase in stress and frustration may occur.

The present study aims to explore the possible social influence on PB and to provide a description of PB characteristics in laying hens housed in visually connected, adjacent units, within a multi-tiered aviary system subjected to two different management practice treatments: continuous (**CON**) and partial (**PARR**) litter access. Originally, we hypothesized a higher frequency of piling behavior in PARR groups compared to CON groups once the litter area became available, due to increased frustration and stress in the animals. Similar responses following restricted litter access have previously been observed for other behaviors, including dust bathing and wing flapping (Bongiorno et al., 2026). However, due to the nature of PB and the potential interdependence among pens, statistical analyzes could not be performed to test this hypothesis.

## Materials and methods

### Birds and husbandry

The Michigan State University Institutional Animal Care and Use Committee reviewed and approved the research procedures ahead of birds’ arrival (PROTO202200159).

A total of 1152 Hy-Line Brown hens hatched in a commercial hatchery, were beak trimmed at 1-day-old and reared on wood shavings in floor units provided with vertical perch access until 16 WOA. At this age, birds were transferred to the Laying Hen Facility at Michigan State University Poultry Teaching and Research Center (East Lansing, MI) and housed in aviary systems provided with wood shavings. The photoperiod was extended from 12L:12D at 16 WOA until it reached 16L:8D at 28 WOA (Hy-Line, 2016). Moreover, a progressive 5-min sunrise consistently occurred at 05.55, while a gradual 45-min sunset occurred before the lights went completely out, the timing of which varied depending on the number of daylight hours. Temperature was kept between 17.8-25 °C by automated heating and ventilation. Feed and water were always available. A visual representation of the timing of feed delivery and feed stimulations is depicted in Fig. 1 as these changed as birds aged and the photoperiod increased. Egg collection was completed before hens gained litter access in PARR and occurred at 1200 in CON groups, in order to collect the eggs laid on the litter in the previous 24 h (Fig. 1). Finally, environmental parameters and health status of the birds were monitored daily by a stock person. More detailed information regarding birds and husbandry is available in Baugh et al. (2026).

**Fig. 1:**
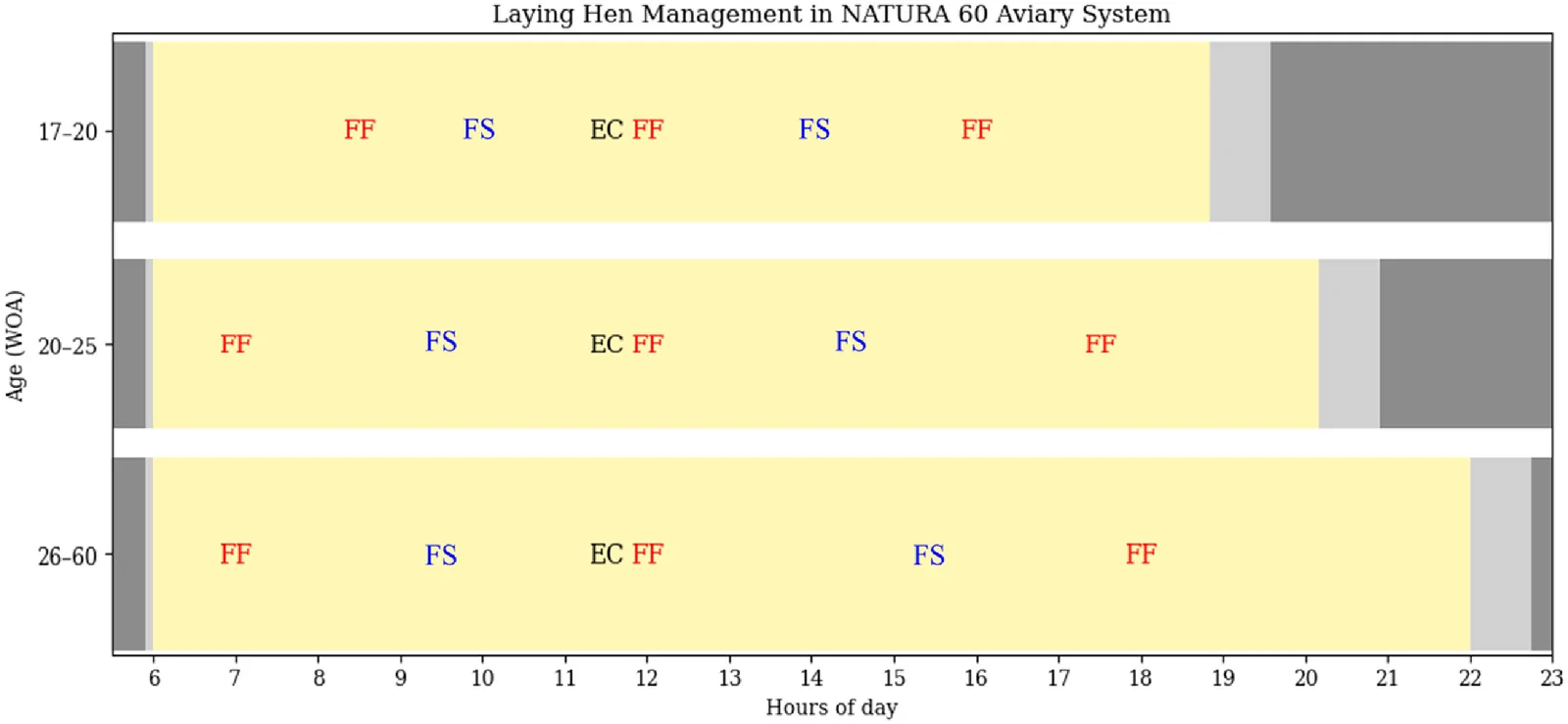
The lighting program, feed distribution, and egg collection procedures. FF=Fresh Feed distribution; FB=Feed Belt run without fresh feed; EC=Egg Collection. Yellow indicates light hours, light grey indicates dawn and dusk, and the dark grey indicates nighttime.

### Configuration of the aviary system and litter access management

The birds in the present study were housed in enclosable, three-tiered aviary systems (NATURA60, Big Dutchman; Holland, MI) (Fig. 2). The stocking density was calculated following United Egg Producers’ guidelines (UEP, 2024) to provide a minimum of 929 cm^2^ of floor space/hen, 15 cm of perch space/hen, and 84 cm^2^ of nest space/hen. Feed was available in the lower and middle tiers, water in upper and lower tiers, and colony nests were located on the upper tier. Perches were provided at all levels, including a perch extending from the lowest level to help birds move to and from the litter. Hens had access to a litter area on one side of the tiers. Further information on the aviary style is provided in Baugh et al. (2026).

**Fig. 2:**
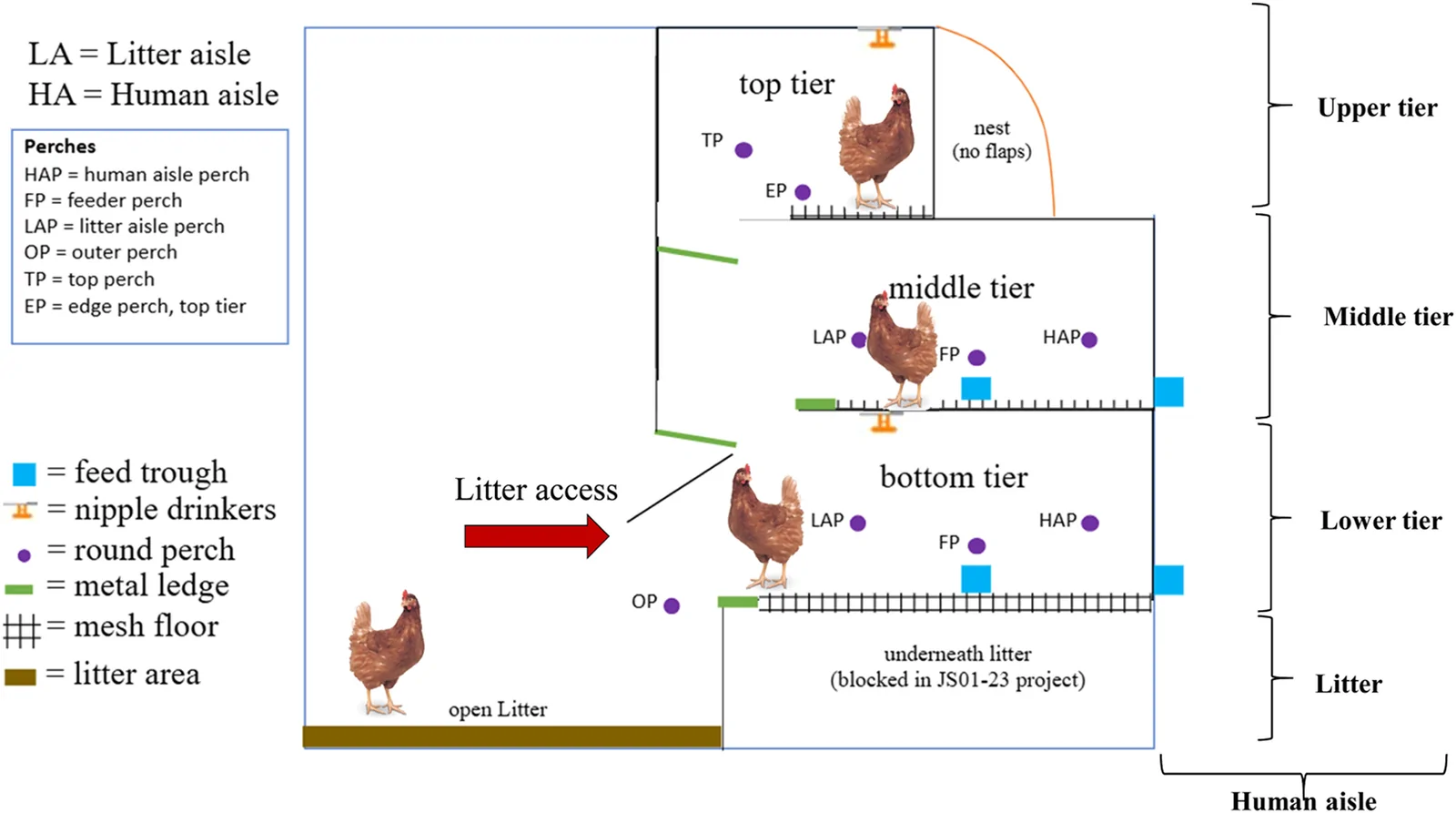
Diagram of NATURA60 aviary system.

The birds were distributed among units in two rooms equipped with this aviary system. Each aviary was subdivided with metal mesh into four units each containing 144 birds, for a total of 576 hens per room. In one room, the birds were confined to the tiered enclosure from 01.00 until 12.00 (24-h format) from 17 to 27 WOA (PARR treatment), while in the other room birds had continuous ability to move between the tiers and the litter area (CON).

### Data collection

Piling was defined as a minimum of ten hens crowded together, with most facing the same direction, often indistinguishable body outline, and no discernible performance of other behaviors (Campbell et al., 2016). The termination of one of these conditions signaled the end of the pile. A combination of bodies, combs, and tails were used for the count. Moreover, if the birds on the outskirts were touching the pile and were oriented towards it, they were considered in the count. Examples of piling events are depicted in Fig. 3.

**Fig. 3:**
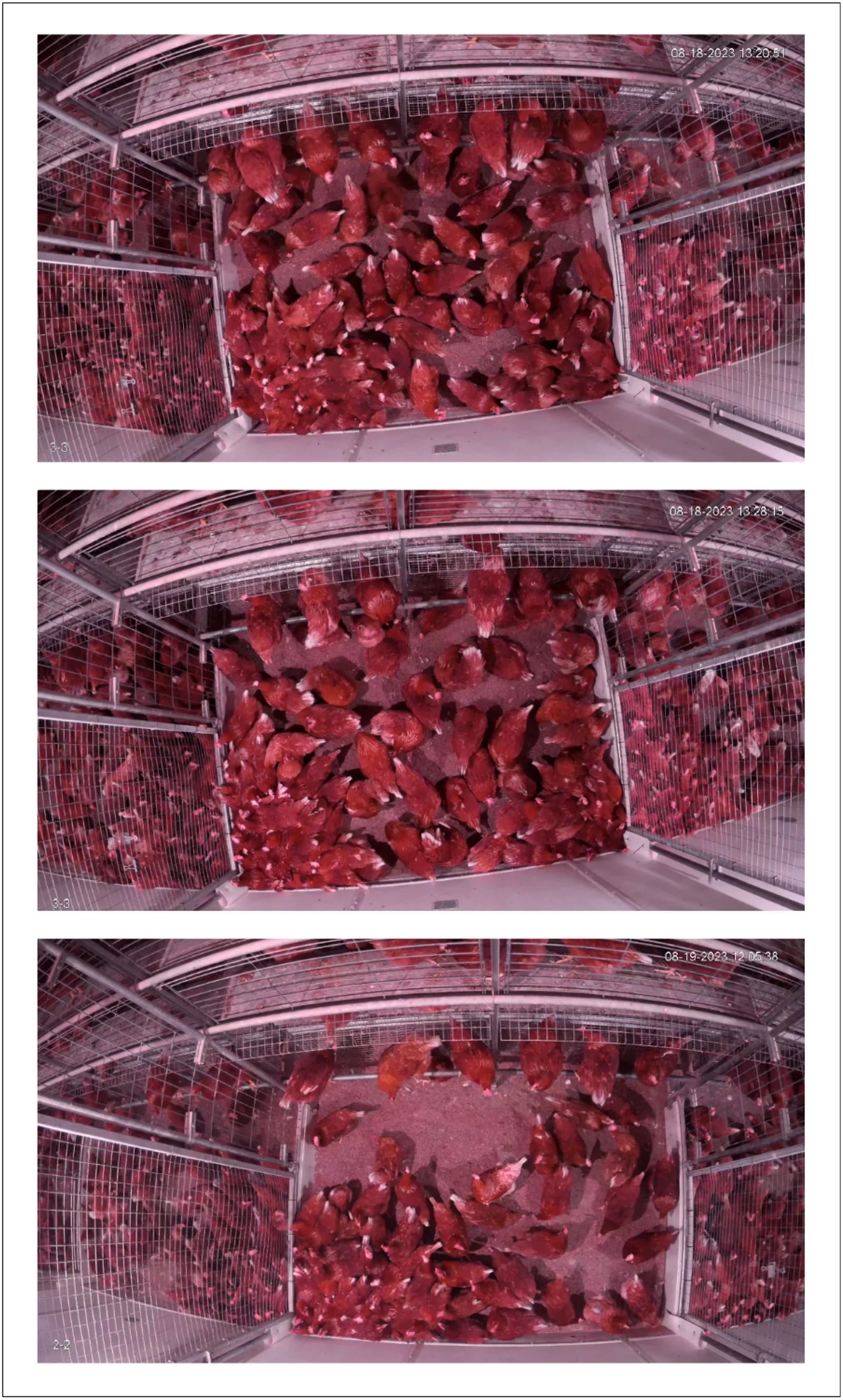
Instances of piling events captured on cameras and included in the video analyses.

Video footage collected at 17, 27, and 44 WOA over a 48-h period was scanned continuously to identify any piling occurrence and the hour in which it occurred was recorded. When a pile changed location within the unit without dispersing and the same group of birds was involved, the event was counted once.

The videos were checked right before PB to determine whether a particular event preceded the piling. If unusual activity was detected immediately before PB began, the video was analyzed backwards at 10-s intervals until the start of unusual activity was identified. This activity was recorded as the preceding event of the pile.

The preceding events were classified as follows:

• *Human presence*, person detected in the unit or corridor and pile oriented toward that human
• *Random*, unidentifiable preceding event (birds performing regular, maintenance-type behaviors immediately prior to PB)
• *Ground pecking*, more than one hen with body cranially tilted toward the ground and/or touching the ground with the beak
• *Other,* e.g., showing interest in the adjacent unit: walking or standing still in alert towards other units (3 events), bird chasing (1 event), sudden bird movement (1 event).

The duration of a PB event (min) was calculated using pile start and end times. Using the duration of the pile, the number of piling birds and total birds on litter were assessed at 1/4, 2/4, and 3/4 of the way through the duration of the pile. As defined above, in order for the event to be considered a pile, a minimum of 10 birds needed to be clustered together; therefore, the number of birds at the beginning and the end of a pile was always equal to 10.

Finally, the position of the pile within the unit (left or right end of the litter area, thus towards the back or the front of the room) was reported, and the activity of each neighboring unit at the beginning of that PB was denoted. Such an activity was categorized as:

• *Status quo*, encompassing regular, maintenance-type behaviors (e.g., dust bathing, foraging, etc.)
• *Agitated/interested toward unit*, encompassing increased birds’ activity and interest shown in the unit in which the pile was occurring
• *Egg collection*, when the stock person was collecting floor-eggs
• *Piling opposite*, when birds were piling in the direction opposite to that of the birds in the evaluated unit
• *Piling toward*, when birds aggregated in the direction of those in the evaluated unit
• *X*, when no adjacent units were present on the left or the right of the investigated unit (which occurred when PB was being examined in units 1 and 4, which were at the end of the row of units).

### Statistical analysis

Video decoding was completed by one observer, who was trained by an experienced ethologist. The training was considered successful when full agreement between the two was achieved. An intra-rater reliability test was performed every other week on the parameters observed on 3 events. Selected video frames were analyzed by the observer to define the accuracy of number of birds in a pile and on litter. The total number of birds on litter, the total number of birds involved in a pile, and the pile duration were examined using an ICC (two-way mixed, absolute agreement), with all results >0.90. The time of preceding event, activity of neighboring units, and position of the pile in the unit overlapped across the observations. To evaluate inter-observer reliability, at the end of the decoding process the most experienced researcher reassessed 10 piling events and their observations compard to those of the observer. Since the possible preceding event observed by the observer was initially not consistent over time, such parameter was reassessed by the most experienced researcher and verified through intra-reliability check (ICC>0.90).

A Monte Carlo simulation (1,000 cases) was performed on the dataset organized by WOA and treatment to determine the probability of obtaining the same number of piling co-occurrences across adjacent units when random combinations of hours were generated. To avoid misleading the simulation result, the events considered were the following:

• events occurring simultaneously in two or more adjacent units when no preceding events were identified.
• events occurring simultaneously in two or more adjacent units, and a preceding event was identified only in one unit.

The dataset was developed in Excel and a Visual Basic for Application used to run the simulations. Per each event, the co-occurrences within the same day, treatment, and WOA, in two adjacent units were counted by attributing to each event a binary code. Two events were declared co-occurrent when starting within the average preceding event + pile duration (min). When presented according to treatment and WOA, the following number of co-occurrences were observed: CON 27 WOA = 14.7; PARR 27 WOA = 21.2; CON 44 WOA = 27.1; and PARR 44 WOA = 23.2. The randomized set of hours was generated accounting for the time window within which the original events occurred according to treatment and WOA. This allowed the time factor to be the only randomized change and did not introduce greater variability than seen in the actual data.

## Results and discussion

The events satisfying the definition of piling regardless of their length were included in the dataset (Table 1). A total of 30, 19, 25, and 21 valid piling events were recorded across 48 h in CON and PARR at 27 WOA, and CON and PARR at 44 WOA, respectively. These piles involved from 10 to 69 birds and lasted from 1.25 to 65.67 min. All PB events were recurring/creeping types of piles. We also observed 26 cases in which birds were showing behavior closely resembling piling. However, in these instances, they did not meet all the requirements necessary to classify them as PB (20 events), or the pile lasted less than 1 min (6 events).

**Table 1:** Descriptive statistics related to pile duration, number of birds piling, # of birds on litter, and % of birds piling in Hi-Lyne Brown hens at 27 and 44 WOA.

	Min		Max		Mean		SD	
**CON – 27 WOA**								
Pile Duration (min)	2		47		17.9		11.5	
# birds piling	17		33		27		5.08	
# birds litter	32		60		42.4		6.00	
% birds piling	29		90		66.3		14.8	
**CON – 44 WOA**								
Pile Duration min		1		66		28.2		15.4
# birds piling		15		39		27.4		5.18
# birds litter		30		52		41.2		6.37
% birds piling		42		87		67.6		11.4
**PARR – 27 WOA**								
Pile Duration min		11		37		22.1		7.94
# birds piling		18		33		26.3		3.79
# birds litter		27		57		38.9		8.05
% birds piling		44		94		70.7		12.7
**PARR - 44 WOA**								
		Min		Max		Mean		SD
Pile Duration min		1		50		20.8		16.7
# birds piling		11		32		24.0		6.22
# birds litter		31		63		41.8		8.27
% birds piling		25		91		60.4		18.7

Descriptive statistics calculated on a total of 30 cases for CON – 27 WOA, 25 cases for CON – 44 WOA, 19 cases for PARR at 27 WOA, 21 cases for PARR – 44 WOA.

PB has been mostly studied in commercial aviaries and floor systems with flocks ranging from 1,200 to about 50,000 birds (Armstrong et al., 2023; Campbell et al., 2016; Jensen et al., 2025; Winter et al., 2021), though one study was performed in experimental settings on replicates of 55 birds each (Winter et al., 2022). Our replicates had a total of 144 birds/each, suggesting the presence of consistent behavioral drivers that are at least partially unrelated to flock size.

No piles were observed at 17 WOA in the present study. Bright and Johnson (2011) reported that panic piling may occur at every stage of rearing or laying, however they observed that creeping/recurring piling was more frequent at 20 and 30 WOA. Besides these observations, no literature has so far focused on the pre-laying period. The absence of PB at 17 WOA in our study may be due to fewer birds on litter compared to later ages. When pullets are reared in systems similar to those they are placed into as hens, improved environmental adaptation with lower fear levels and discomfort is observed (Nicol, 2020; Du et al., 2022). Since our experiment used floor-raised birds, their unfamiliarity with a aviary-tiered system likely led to a low number of birds counted on litter and may explain the absence of PB right after transferring birds from the rearing to the hen facility. However, further research is required on PB patterns in aviary-raised pullets to have a better understanding of the impact of age versus housing characteristics on PB behavior. Interestingly, Winter et al. (2022a) observed PB in experimental settings with bird litter crowdedness similar to the one we had in the present study at 17 WOA. However, we did not see PB despite similar litter density. Since Winter and colleagues used white birds during their laying period, while we had brown birds at 17 WOA, both genotype and WOA may have played a role in the absence of PB.

Table 1 provides descriptive statistics of piling duration and number of birds involved. Pile duration ranged from 17.9 ± 11.5 min in CON at 27 WOA to 28.2 min ± 15.4 in CON at 44, with intermediate values for PARR birds. Moreover, the percentage of birds involved in the piles was not consistent. The variation of duration and birds involved in a pile is in line with what observed in previous studies conducted in both experimental settings (Winter et al., 2022a) and commercial farms (Winter et al., 2022b), underlining the unpredictability of the behavior.

Table 2 depicts the daily distribution of piling events across treatments and ages. PARR birds did not have access to litter at 27 WOA, thus PB could only occur in the afternoon. At 27 WOA, 13.3% of PB events were observed in CON groups before 12.00, and when PB through the day was considered, a total of 30 piles were observed in CON compared to 19 in PARR. These results suggest that the total amount of time hens spend on litter may affect PB frequency. Despite reducing the number of piling events, restricting litter access may also negatively impact animal welfare, as several welfare-relevant behaviors cannot be performed (e.g., foraging, dust bathing, and wing flapping), thus we do not recommend it as PB mitigation strategy. However, in PARR at 44 WOA 10% and 91% of the events were observed 4 - 8 and 8 - 16 h after lights on, respectively. This distribution is similar to what was reported in previous studies in brown and white flocks, where most of the events occurred 4-5 to 8-10 h after lights on (Herbert et al., 2021; Winter et al., 2021; Winter, Stratmann, et al., 2022). Nevertheless, in CON at 44 WOA, 12% of the events were observed between 0 - 4 h after lights on, while 48 and 40% were seen at 4 - 8 and 8 - 16 h after lights on, showing a more even distribution than previous research. PB at 44 WOA has never been described in literature before, and it is not clear why PARR birds showed similar PB daily frequency to reports from previous studies while CON hens did not.

**Table 2:** Daily frequency (blocks of 4 h over 16-h light period) of piling events occurred in Hi-Lyne Brown hens at 27 and 44 WOA.

Daily Frequency of Piling Events	
h after lights on	% piles in range
**CON – 27 WOA**	
0 - 4	3
4 - 8	50
8 - 16	47
**CON – 44 WOA**	
0 - 4	12
4 - 8	48
8 - 16	40
**PARR – 27 WOA**	
0 - 4	0
4 - 8	47
8 - 16	53
**PARR – 44 WOA**	
0 - 4	10
4 - 8	91
8 - 16	0

A pie chart depicting the preceding event occurrence for each treatment and WOA is provided (Fig. 4). Overall, few preceding event categories were identified at 27 WOA (Fig. 4A,B), while a broader variety was identified at 44 WOA (Fig. 4C,D) despite the overall similar number of piling events recorded. As a similar number of piling events occurred across weeks in CON and PARR (30 *vs* 25 events, in CON at 27 WOA and 44 WOA, respectively; 19 *vs* 21 events in PARR at 27 and 44 WOA, respectively) even when no clear preceding event was identified, the behavior may be driven by an unidentified internal motivation rather than the presence of external factors. However, random events preceded approximately 30% of piling events in PARR hens compared with about 60% in CON hens. Together with the occurrence of cracked eggs and ground pecking as preceding events, this pattern may suggest that piling in PARR hens was more frequently associated with observable motivations related to foraging and/or exploration, potentially resulting from restricted litter access. Similar behavioral responses to deprivation of litter-related activities have been reported previously (Vestergaard, 1982; Nicol, 1987; Bongiorno et al., 2026). However, given the limited sample size, these findings should be interpreted cautiously, and no definitive conclusions can be drawn.

**Fig. 4:**
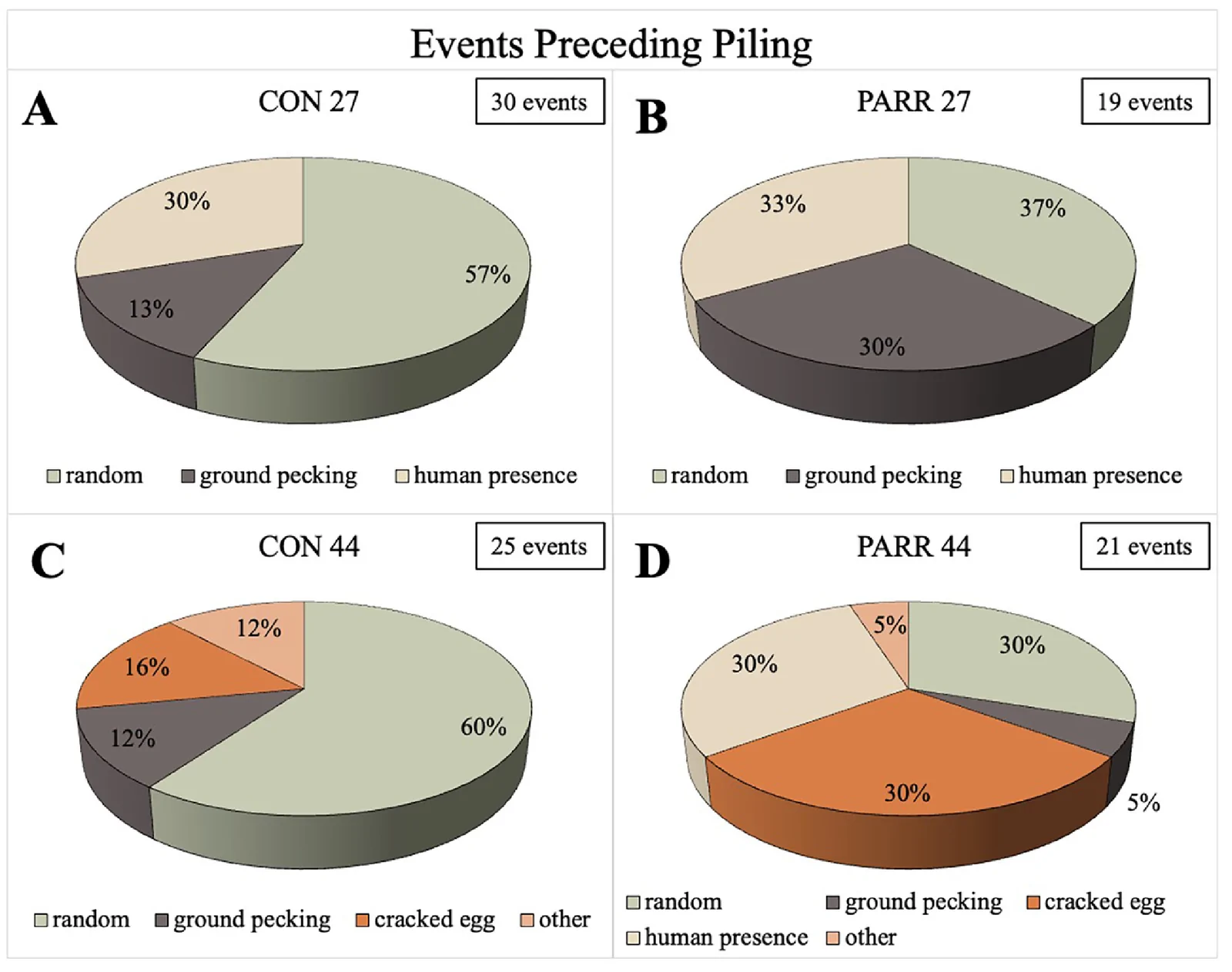
Pile preceding events of Hy-Line brown hens divided by treatments (CON=continuous litter access; PARR=partial litter access) and weeks of age (27, 44 WOA).

Of the total piling events observed, 43% (CON27), 63% (PARR27), 76% (CON44), and 52% (PARR44) occurred simultaneously in two or more units, and for the majority of these there was no obvious preceding event. Screening the PB events in this way allowed us to isolate events that were possibly linked to social influences rather than management/environmental factors in common among all the units (e.g., human presence due to egg collection). The piling occurrences that clustered together are shown in Fig. 5.

**Fig. 5:**
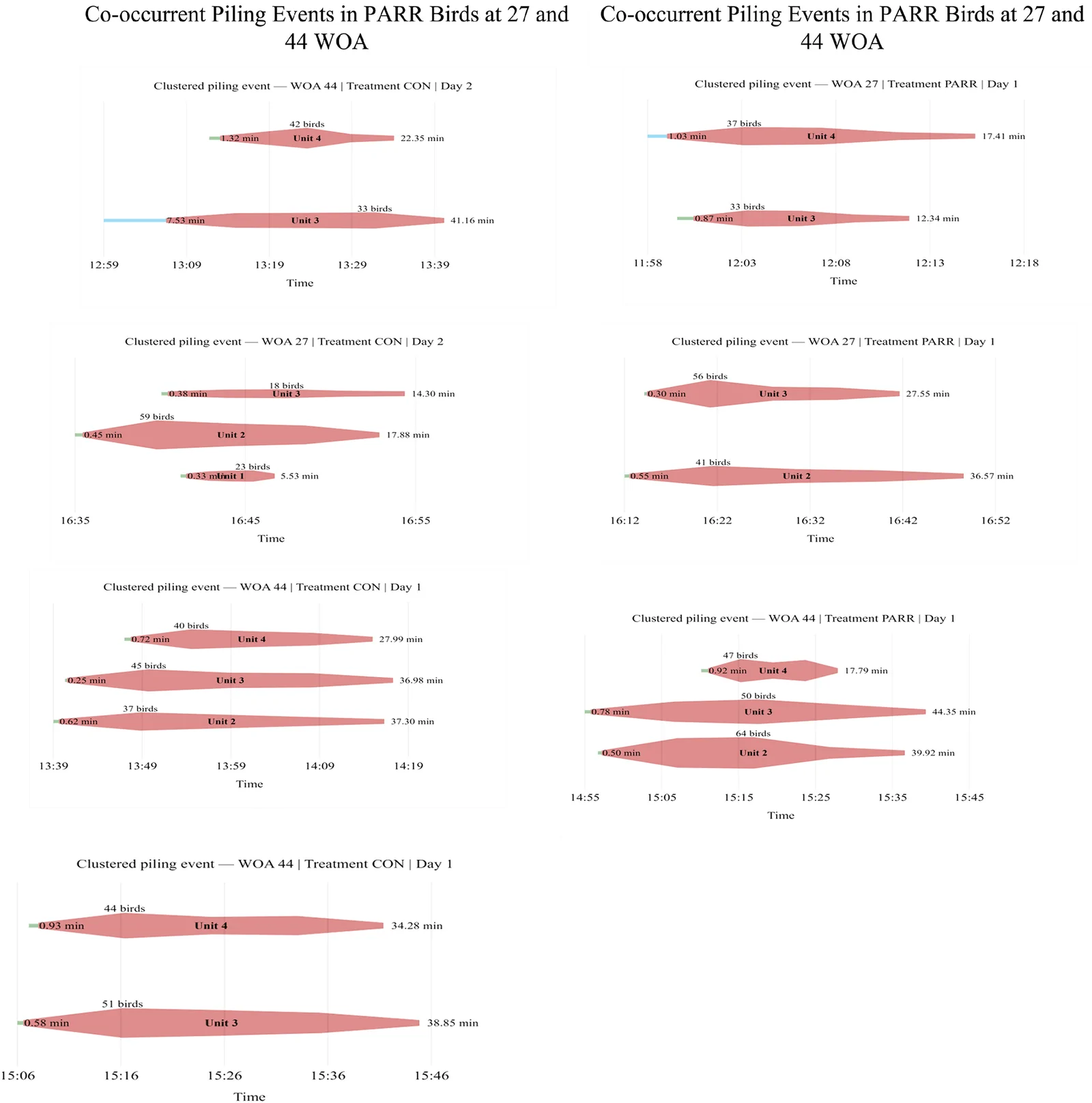
Co-occurrent piling events used in Monte Carlo simulation in Hy-Line brown hens divided by treatments (CON=continuous litter access; PARR=partial litter access) and weeks of age (27, 44 WOA).

It is interesting to notice that the first pile initiated within a pile cluster always lasted the longest, regardless of treatment or WOA. This suggests that birds involved in the first pile of co-occurring events may have been driven by greater motivation/or experienced to a stronger stimulus that led to PB, while the other co-occurring piles may show a socially facilitated response. Previous studies have reported the existence of facilitation response in birds (Hoppitt et al., 2007; Collins et al., 2011), and this phenomenon has previously been considered as a possible explanation of why laying hens face the same direction within a pile (Winter et al., 2021). To examine whether social facilitation could be responsible for the nearly synchronous piles occurring in >1 unit in a room at the same time when no apparent preceding events (random) were present, Monte Carlo simulations were performed. Monte Carlo simulation adopts random sampling and statistical modeling to predict the probability of observing certain outcomes (Harrison et al., 2010; Kissell, 2021). The Monte Carlo method has been used previously to understand piling (Armstrong et al., 2023; Barrett et al., 2014; Gray et al., 2025) though not to examine social influence. However, Witham (2018) explored social relationships between pairs of monkeys, where Monte Carlo shuffling was adopted to determine which pairs of monkeys associated with each other at a greater frequency than expected by chance alone. As we selected piling event clusters occurring in response only to all random (unidentified) stimuli, or all random stimuli except one, significant Monte Carlo results would have pointed towards social facilitation as more co-occurrences would have been observed in our dataset than what would be generated by chance. However, no significant effect of social influence was detected, suggesting that piling may be occurring simultaneously in adjacent units due to other external factors that were not detected by human observers (or that were possible to observe from a video recording). For instance, Keeling and colleagues (2017) showed that some gregarious behaviors may be related to resource use rather than a social facilitation response, and this hypothesis needs to be further investigated.

Fig. 6 shows the number of events in which 0 to 25, >25 to 50, >50 to 75, and >75 to 100% of birds on litter were involved in a pile. Interestingly, a greater number of events involved >50% to 100% of birds on litter while only a few involved 0 to 25%. These findings may indicate the presence of a facilitated response within a flock, although genetic similarity may also play a role in sudden bird clustering during a piling event. Commercial hybrids present specific phenotypical and behavioral traits that can vary across hybrids such as different levels of fearfulness and stress (Nelson et al., 2020), as well as in their resource use and spatial distribution (Ali et al., 2016; Villanueva et al., 2017). Decision-making processes in gregarious birds can also be affected by the degree of uniformity of personalities within a flock. This leads to faster decision-making and thus to homogeneity of behavioral performance in highly uniform groups of birds (Carere et al., 2025).

**Fig. 6:**
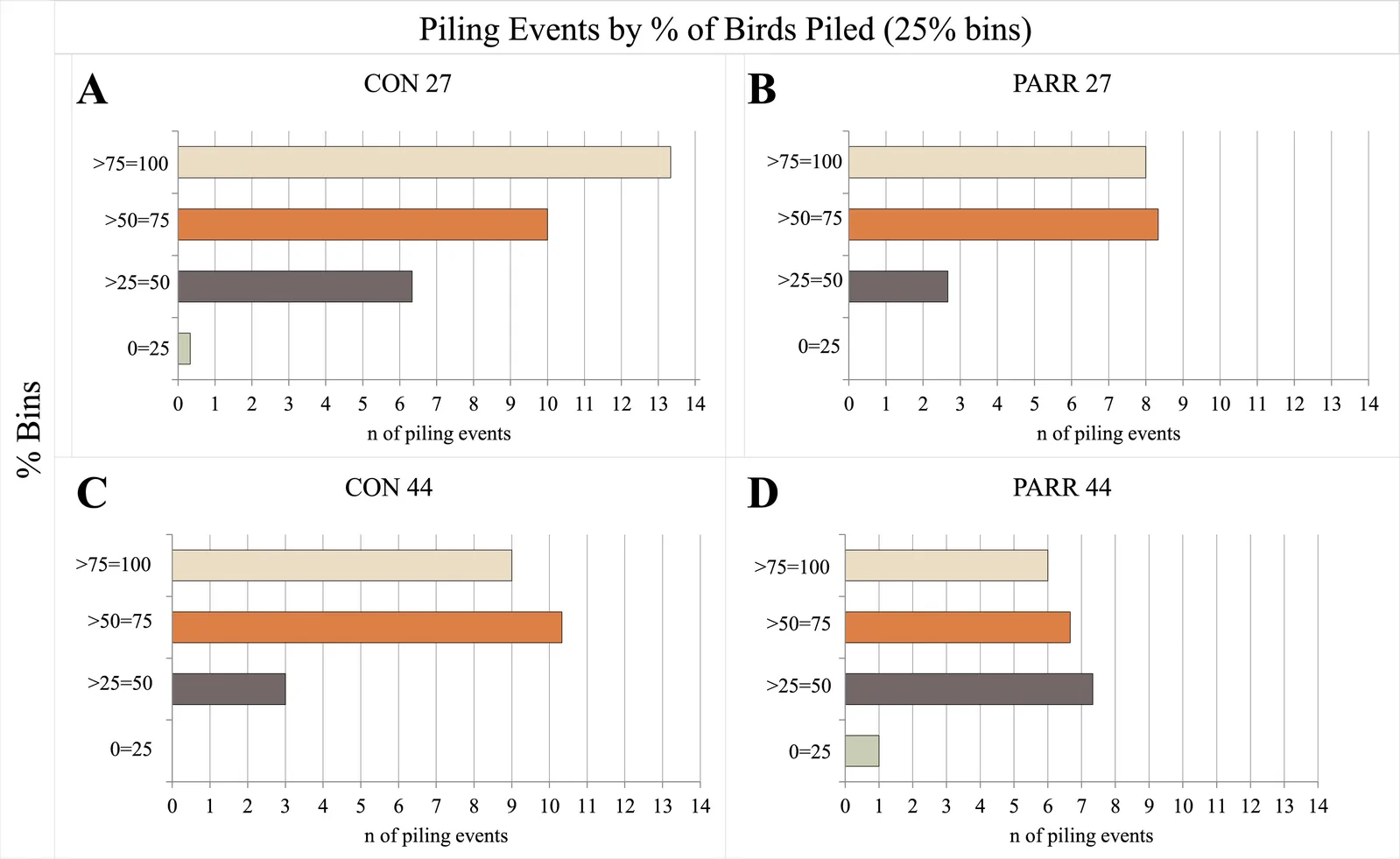
Number of piling events in Hy-Line brown hens in which the percentage of piling birds (relative to birds on the litter) fell within 0–25%, 25–50%, 50–75%, or 75–100%, measured at 0.25, 0.50, and 0.75 of pile duration, and divided by treatments (CON = continuous litter access; PARR = partial litter access) and week of age (27 and 44 WOA).

Finally, Fig. 7 depicts the activity of units adjacent to unit 2 (Fig. 7A) and 3 (Fig. 7B) at the onset of piling events across treatments and WOA. Units 2 and 3 were selected for the analysis as they had adjacent units on both right and left side and were less affected by external system interferences due to their location in the center of the row of units. Moreover, these graphs are presented separately to point out the intrinsic variability of the data across units despite homogeneous genetics, physical environment, and management. Overall, the predominant activity recorded in the adjacent units at the onset of a piling event in focal units 2 or 3 corresponded to *status quo*, while low percentages were recorded for birds piling near or away from the focal unit. On one hand, these findings are in line with the absence of facilitation response shown by our Monte Carlo simulation and suggest that PB observed in units 2 and 3 were not likely responsible for initiation of clusters of piles in times across units in a room. However, our evaluation focused only on the onset of the focal pile and does not exclude the occurrence of PB in adjacent units at any other point during the duration of the focal pile.

**Fig. 7:**
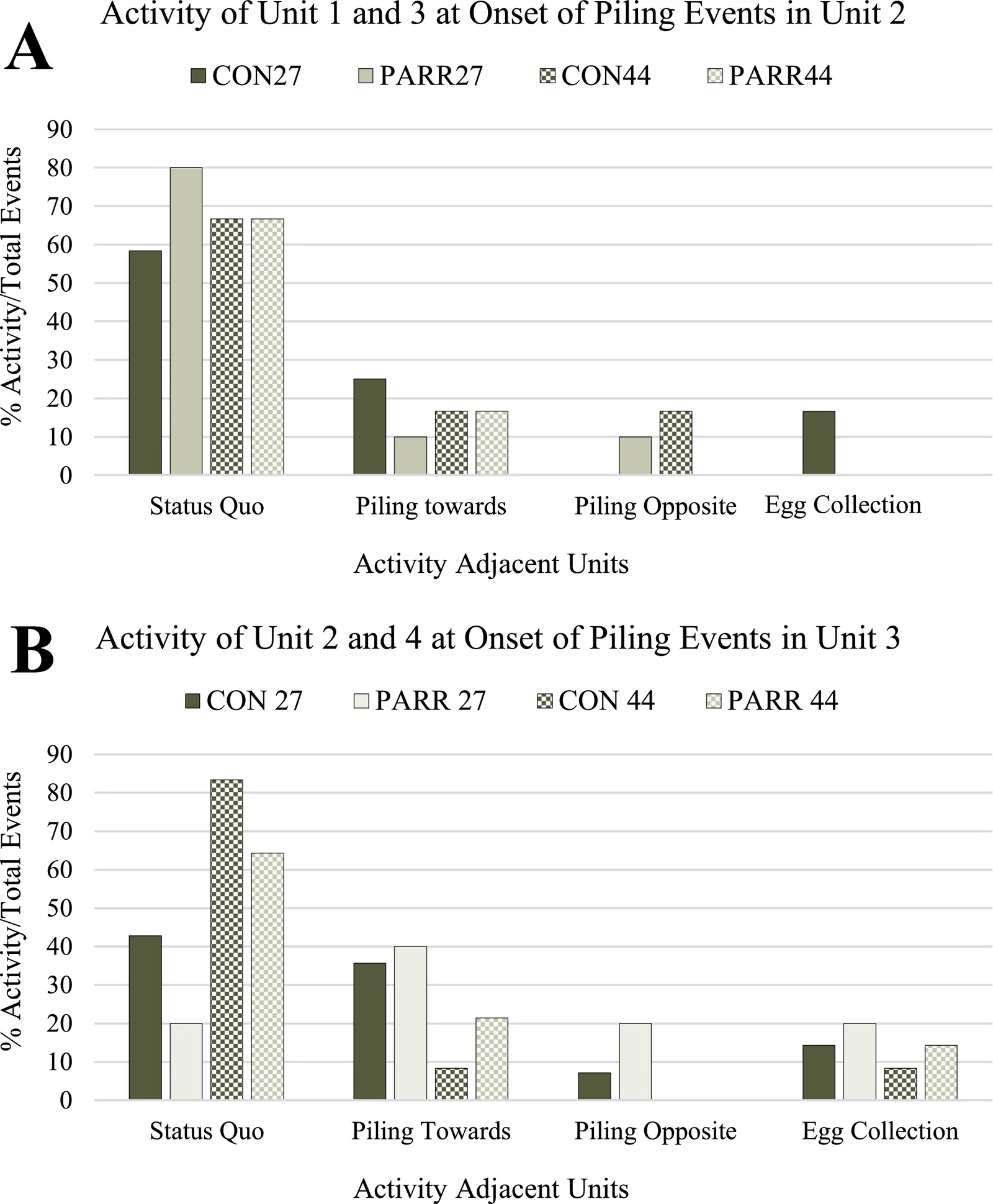
Activity of Hy-Line brown hens monitored in units adjacent to unit 2 (Fig. 7A) and 3 (Fig. 7B) at the onset of piling events. Piling events from units 2 and 3 were considered, as they had birds on both left and right side. Activity is displayed by treatments (CON = continuous litter access; PARR = partial litter access) and week of age (27 and 44 WOA).

## Limitations and further research

The present study offers a description of PB under different litter management strategies across different ages in one commercial strain of brown laying hens. Overall, the experimental settings favored the evaluation of social influence on PB across units. However, the same experimental constraints did not allow statistical comparisons between litter management strategies and WOA, as the independence of replicates could not be assured. Moreover, rigorous criteria were adopted for the selection of piling event clusters in the dataset used for Monte Carlo simulation. On one side, such selection avoided simulation biases due to factors simultaneously affecting all the units within a room. On the other side, this significantly reduced the size of the dataset used for Monte Carlo simulation. For this reason, further research with a larger sample size is required to better explore both social facilitation in PB and the effects of litter management treatments and bird age. Finally, this study focused on one brown hen strain, and further investigation involving different strains is required to better understand PB implications and its effects on a commercial scale.

## Conclusions

In conclusion, this study provides useful descriptive insight into the effect of litter management strategies and WOA on PB, as well as a first evaluation of possible social facilitation influences, laying the foundations for future investigations with a larger sample size. Overall, PB may be mainly driven by factors related to the laying period, as no piling events were observed during the rearing phase. Moreover, hen age may affect the variety of external stimuli triggering PB, although internal motivation may also be involved. In fact, a similar number of piling events across groups was detected regardless of the variety of identified preceding event stimuli. Finally, Monte Carlo simulation did not show statistical significance, suggesting the absence of social facilitation in the co-occurring cases observed in the current study.

## Declaration of AI and AI-assisted technologies in the writing process

During the preparation of this work, the authors used ChatGPT (OpenAI) to assist with the implementation and troubleshooting of the Monte Carlo simulation and statistical programming. After using this tool, the authors reviewed and edited the content as needed and take full responsibility for the content of the publication.

## Author Contributions

All three authors meet the four requirements for authorship required by Poultry Science by 1) providing substantial contributions into the conception or design of the work (VB, JS, and AA) as well as acquisition (VB), analysis (VB, JS), and interpretation of data (VB, JS, AA); 2) drafting (VB, JS) or reviewing the manuscript (AA), 3) giving final approval to the exact, final version of the manuscript (VB, JS, and AA) and 4) agreeing to be personally and publicly responsible for all aspects of the work and ensuring that any questions regarding the accuracy or integrity of the research are thoroughly investigated and resolved (VB, JS, and AA).

## Disclosures

Ahmed Ali reports financial support was provided by National Institute of Food and Agriculture. Janice

Siegford reports financial support was provided by Michigan Alliance for Animal Agriculture. Janice

Siegford reports financial support was provided by National Institute of Food and Agriculture. J.

Siegford serves as an unpaid member of the United Egg Producers Scientific Advisory

Committee. No support or endorsement was received from United Egg Producers for this work.

If there are other authors, they declare that they have no known competing financial interests or personal relationships that could have appeared to influence the work reported in this paper.
